# A green approach to obtain stable and hydrophilic cellulose-based electrospun nanofibrous substrates for sustained release of therapeutic molecules†

**DOI:** 10.1039/c9ra03399h

**Published:** 2019-07-09

**Authors:** Manja Kurečič, Tamilselvan Mohan, Natalija Virant, Uroš Maver, Janja Stergar, Lidija Gradišnik, Karin Stana Kleinschek, Silvo Hribernik

**Affiliations:** Laboratory for Characterization and Processing of Polymers, Faculty of Mechanical Engineering, University of Maribor Smetanova 17 2000 Maribor Slovenia tamilselvan.mohan@um.si manja.kurecic@um.si +386 2220 7902; Faculty of Electrical Engineering and Computer Science, University of Maribor Koroška Cesta 46 SI-2000 Maribor Slovenia; Institute of Biomedical Sciences, Faculty of Medicine, University of Maribor Taborska Ulica 8 2000 Maribor Slovenia

## Abstract

Stable and (bio)-compatible nanofibrous matrices showing effective incorporation and release of nonsteroidal anti-inflammatory drugs (NSAIDs) hold a huge potential in tissue regeneration and wound healing. Herein, a two-step, water-based and needleless electrospinning method is used to fabricate thermally cross-linked multifunctional nanofibrous substrates from a hydrophilic cellulose derivative, *i.e.* carboxymethyl cellulose (CMC), and polyethylene glycol (PEG) with an *in situ* incorporated NSAID, diclofenac (DCF). Electrospun bi-component blend nanofibers, strongly linked together by ester bonds, with different degrees of cross-linking density are achieved by varying the concentrations of butanetetracarboxylic acid (BTCA, a green polycarboxylic cross-linker) and the sodium hypophosphite (SHP) catalyst, and the temperature. The results demonstrated that not only the dimensional stability and swelling properties could be better controlled but also the morphology, fiber diameter, surface area, pore volume, pore size, and functionality of the cross-linked nanofibers. Release kinetics of DCF from the nanofibrous substrates are controlled and prolonged up to 48 h, and the overall released mass of DCF decreased linearly with increasing cross-linking degree of BTCA and SHP. Fitting of release data using various kinetic models revealed that the release of DCF follows a non-Fickian (diffusion and erosion controlled) to Fickian mechanism (only diffusion-controlled process). Cell viability testing based on crystal violet dyeing showed that the DCF-incorporating nanofibers have excellent biocompatibility and no toxic effect on human skin fibroblast cells. Overall, the reported DCF-incorporating nanofibrous substrate demonstrates high potential to be used as a smart drug delivery system in wound healing, especially due to its noninvasive characteristics.

## Abstract 2

Stable and (bio)-compatible nanofibrous matrices showing effective incorporation and release of nonsteroidal anti-inflammatory drugs (NSAIDs) hold a huge potential in tissue regeneration and wound healing.

## Graphical abstract

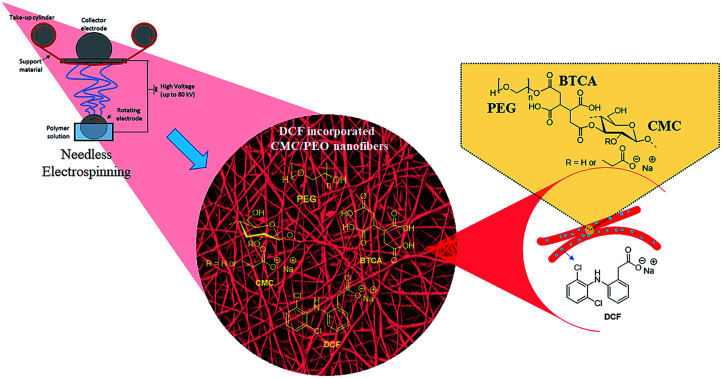

## 1. Introduction

Nanofibers of polymeric materials have been in focus for several years because of their unique morphology and internal architecture. Due to their superior features such as high surface area-to-volume ratio, nanoscale diameter, porous structure, and flexibility over other forms of the same materials, polymer nanofibers are used in several areas of applications, including packaging, electronics, membranes, air filtration, dye scavenging, semiconductors, *etc.*^1–7^ In addition, they hold huge potential in wound healing, especially in cell growth, owing to their resemblance to extracellular matrices (ECM) and extracellular macromolecules such as collagen.^8^ Apart from cell adhesion and proliferation, polymer nanofibers have been used for the release of various therapeutic molecules, including proteins, peptides, DNA, and drugs.^9–12^ Consequently, numerous materials, active ingredients, and polymers (synthetic and natural) were exploited as potential drug delivery systems for the treatment of several diseases including cancer,^13^ chronic wounds,^10^ periodontal disease,^14^*etc.* However, control over the rate and quantity of drug release is critical for successful treatment of specific wound type (*e.g.*, chronic). In this context, a plethora of studies on fabrication of drug incorporated polymer nanofibers were performed and proven to be a promising sustained and smart drug delivery system during the last years.^15–17^ Needless electrospinning technique is commonly used to obtain the mentioned ultrafine fibers (micro to nano-meter diameters) in larger quantities (10 g h^−1^) from polymeric solution or polymer blends containing active drug molecules.^18^ Even though stable nanofibers blends can be easily made from hydrophobic (*e.g.*, cellulose acetate,^19^ polycaprolactone,^20^*etc.*) or mixture of hydrophobic and hydrophilic polymers (*e.g.*, poly(lactic-*co*-glycolic acid) (PLGA)/polyethylene glycol (PEG),^21^*etc.*), they are not suitable for incorporation and release of water soluble drug such as diclofenac (DCF), the nonsteroidal anti-inflammatory drug (NSAID), which is often used for the treatments of pain and inflammatory diseases.^22^ As a result, the blended nanofibers from several hydrophilic polymers such as chitosan, gelatin, carboxymethyl cellulose (CMC), polyethylene glycol (PEG), silk, cellulose nanocrystals, collagen, *etc.* have been studied and reported elsewhere.^15,23,24^

Amongst several others, CMC, a derivative of cellulose (naturally occurring biopolymer), is often used over synthetic polymers in wound healing applications for the controlled release of drugs, due to its hydrophilicity, biocompatibility, extensive fluid uptake capacity, degradability, and inexpensiveness. However, CMC alone provide challenges in electrospinning, which can be overcome by incorporation of hydrophilic, non-immunogenic, and non-toxic spinning agent such as PEG. The latter provides adequate chain entanglement in the liquid state, and thus resulting in uniform fiber formation. Even though the blends of CMC and PEG are electrospinnable,^25^ the hydrophilic nanofibers substrate still possess some disadvantages; as they can be rapidly dissolved/destroyed upon contact with wound liquid environment, preventing controlled and prolonged drug release. Thus, besides the fluid uptake capacity, certain dimensional stability of the nanofibrous materials in the wound-mimicking environment must be ensured. One way to resolve this issue is the usage of chemical cross-linkers such as aldehydes, carbodiimides,^26^ epichlorohydrin, diglycidyl ether, glycidylmethacrylate, *etc.*^27^ However, these cross-linkers have limited applications in wound healing, due their toxicity towards human skin cells. Therefore, multifunctional polycarboxylic acid (*e.g.* 1, 2, 3, 4 butanetetracarboxylic acid, BTCA), which is eco-friendly, water soluble, and non-toxic, is suitable candidate for cross-linking of CMC/PEG nanofibers. While the CMC/PEG based hydrogels have been used for drug delivery and wound dressing/healing applications,^28,29^ the systematic and detailed studies on electrospun nanofibers from the same polymers for the release of DCF and growth of human skin cells such as fibroblast are very few. Especially, no studies can be found on the thermal cross-linking and stabilization of CMC/PEG nanofibers with BTCA through a sustainable, greener and two-steps approach.

The aim of our study was therefore to prepare water insoluble CMC/PEG nanofibrous substrates for the controlled and prolonged delivery of DCF. To achieve this, the DCF incorporated CMC/PEG blend aqueous solution was mixed with BTCA and SHP at different concentrations. The viscosity, conductivity and surface tension of these solutions consisting of DCF or no DCF were investigated in detail before electrospinning. The electrospun biocomponent CMC/PEG nanofibers incorporated with and without DCF were characterized in terms of chemical composition, morphology, pore size, pore volume, fiber diameter as a function of BTCA and SHP concentration and (cross-linking) temperatures by using several modern analytical tools. The crosslinking efficiency of BTCA and its impact on fibre swelling capacity and stability was investigated by conventional swelling and quartz crystal microbalance with dissipation (QCM-D) measurements, respectively. *In vitro* release kinetics of DCF, at physiological pH, from nanofibrous substrates cross-linked with different BTCA concentration were performed, and the release data was best fitted to different kinetic models. Prepared nanofibrous substrates were also tested in regard of their potential applicability in biomedical applications. This was done by determining the cytotoxicity of drug incorporated nanofibrous substrates towards human skin derived fibroblasts. The obtained results from these studies should lay a strong basis; which could be potentially used to build multifunctional and stable nanofiber system for the targeted delivery of several therapeutic agents in the field of wound healing, and tissue repair, in general.

## 2. Experimental section

### 2.1 Materials

Sodium salt of carboxymethylcellulose (CMC, *M*_w_ = 90 kDa, DS_COOH_: 0.7) and poly(ethylene oxide) (PEG, *M*_w_: 600 kDa) were purchased from Acros Organics (USA). 1,2,3,4-Butanetetracarboxylic acid (BTCA) was purchased from Merck (USA). Sodium hypophosphite monohydrate (SHP, NaPO_2_H_2_·H_2_O) and diclofenac sodium salt (DCF) were purchased from Sigma-Aldrich (Germany). Quartz crystal microbalance (QCM) crystals coated with gold layer (QSX301) were purchased from LOT-Oriel, Germany. Milli-Q water from a Millipore water purification system (Millipore, USA; resistivity = 18.2 MΩ cm at 25 °C) was used for all sample preparation.

### 2.2 Preparation of samples for electrospinning

5 g of CMC was added to 95 g of water (5 wt%) and stirred with a 3-bladed mechanical propeller stirrer (Ika, Germany) at 2000 rpm until a homogenous solution was obtained (∼24 h). In the same way, 5 wt% PEG solution was prepared. The cross-linker BTCA and the catalyst SHP at four different concentrations (3, 5, 7 and 10 wt% – based on the weight of CMC) were prepared by dissolving appropriate amount of BTCA or SHP in MilliQ water. The above prepared four types of solutions (CMC, PEG, BTCA and SHP) were then used for creating formulations for electrospinning. Briefly, CMC (5 wt%) and PEG (5 wt%) solutions were mixed in a 1 : 1 ratio. To this, 1 ml of BTCA and SHP, both prepared at four concentrations (3, 5, 7 and 10 wt%), were added and stirred for 1 h. In total 5 samples were prepared, and they are designated as BTCA_0%, BTCA_3%, BTCA_5%, BTCA_7% and BTCA_10%, respectively. The control sample BTCA_0% *i.e.* CMC/PEG contains no BTCA and SHP. To these five solutions, DCF (10 wt%, based on the weight of CMC, dissolved in water) was added and stirred with a 3-bladed mechanical propeller at 2000 rpm for 1 h. These new five samples prepared with DCF were designated as DCF_BTCA_0%, DCF_BTCA_3%, DCF_BTCA_5%, DCF_BTCA_7%, and DCF_BTCA_10%, respectively.

Prior to electrospinning, all these ten prepared solutions were characterized in regard of their viscosity, surface tension and conductivity using viscometer (Fungi lab), photogoniometer (OCA35 Data Physics) and conductivity meter (Mettler Toledo), respectively.

### 2.3 Needless electrospinning

Electrospinning of all solutions was conducted using a pilot-scale needless electrospinning apparatus ElMarco Nanospider NS LAB 500 (Czech Republic). In comparison to widespread single needle electrospinning setup, with low production rate 0.1–1 g h^−1^,^30^ the electrospinning equipment used in this study, has the production rate of up to several 10g h^−1^. Nanospider is a needle-less electrospinning apparatus with a high voltage power supply (up to 80 kV), feeding unit (a bathtub with rotating electrode – cylinder or wire) and a grounded collector (cylinder or wire electrode). In the electrospinning process, we used stainless steel cylinder electrode. All nanofibrous samples were electrospun at constant conditions; distance between the electrode and the collector plate set at 17 cm, accelerating voltage at 60 kV, time of electrospinning was 40 min, while temperature and humidity of working environment were set at 20 °C and 30%, respectively. Electrospun nanofibers were collected on a 100% polypropylene (PP) nonwoven fabric (Pegatex® S), used as a support material, manufactured with spun bond technology and supplied by Pegas nonwovens s.r.o. (Znojmo, Czech Republic).

For electrospinning on QCM Au-crystals, a specially designed sample holder made from Teflon was used and mounted together with the QCM crystals on the PP fabric.^31^ The role of the sample holder is to avoid deposition of nanofibers at the edges and the backside of the crystal. For the electrospinning experiments, the same parameters as mentioned above were used, but the electrospinning of solutions were performed for 10 s.

#### Crosslinking of electrospun nanofibers

All the electrospun samples were peeled-off from the PP support and cut into 6 cm × 6 cm. The QCM crystals with nanofibers attached to the fabric were also removed. Subsequently, they were transferred to a glass Petri dish and dried in an oven at 80 °C for 5 min. Then they were kept at three different temperatures (120 °C, 140 °C, 160 °C) for 15 min for the cross-linking of nanofibers.

### 2.4 Scanning electron microscopy (SEM)

The morphology of electrospun nanofibers before and after crosslinking was analysed by Field Emission Scanning Electron Microscopy (FESEM), by using a Carl Zeiss FE-SEM SUPRA 35 VP electron microscope. The images were recorded with an acceleration voltage of 1 keV at approximately 4.5 mm working distance. Electrospun samples were fixed on a conductive carbon tape attached to a metal microscope holder and sputtered with a thin layer of palladium using a Benchtop Turbo sputtering device (Denton Vacuum, USA). The average diameter of cross-linked and non cross-linked nanofibers was measured directly from selected SEM images using ImageJ software and is given as an average value for each sample calculated from at least 20 measurements.

### 2.5 Attenuated total reflection-Fourier transform infrared (ATR-FTIR) analysis

The ATR-IR spectra of nanofibrous samples were measured using a PerkinElmer FTIR System Spectrum GX Series-73565 at a scan range of 4000–650 cm^−1^. A total of 32 scans were performed at all measurements with a resolution of 4 cm^−1^.

### 2.6 Swelling studies

A weighed amount of dry electrospun nanofibrous substrates was put in sphere lattice (sieve pouch) and immersed in water at 37 °C for 24 h. After swelling, nanofibrous substrates were taken out, left to drain for 1 min and wiped with filter paper to remove surface bound water and weighed again. Obtained sample weights were used to determine the water absorption capacity (WAC) swelling degree by the following equation:1
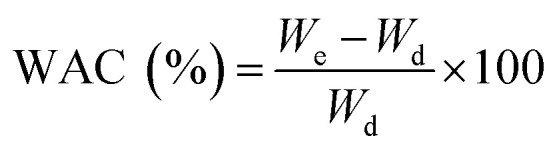
where WAC is the equilibrium water absorbency (%), *W*_e_ and *W*_d_ are the weight of swollen and dry nanofibrous substrates, respectively.

### 2.7 Porosity determination

Nitrogen adsorption/desorption measurements were performed on a Micromeritics TriStar II 3020 porosimeter (Micromeritics, USA) using a Brunauer–Emmett–Teller (BET) model for surface area evaluation. All samples (before and after crosslinking) were cut into small pieces and degassed with nitrogen gas at 40 °C prior to the measurements to remove volatile compounds. Average values of BET measurements were calculated from three repetitions of each sample measurement.

#### Statistical analysis

All numerical values are given as mean ± SD. Statistical analysis was performed using SPPS Statistics 25 (IBM Corp. Armonk, NY, USA). A one-way ANOVA followed by a post-hoc Bonferroni test was carried out. *P*-values < 0.05 were considered statistically significant.

### 2.8 Quartz crystal microbalance with dissipation (QCM D)

Adsorption as a function of the resonance frequency change of an oscillating quartz sensor was monitored using the QCM-D technique (model E4 from Q-Sense, Gothenburg, Sweden). The QCM-D instrument determines changes in frequency (*f*) and dissipation (*D*) of an oscillating quartz crystal. Deposition of mass or changes in the rigidity of material on the crystal surface can be detected. Negative frequency shifts (*f*) indicate a deposition of mass whereas positive dissipation shifts (*D*) are caused by a reduced rigidity of the coating. The QCM-D measurements were conducted at the fundamental frequency of 5 MHz and its overtones. A detailed description of the QCM-D technique can be found elsewhere.^32,33^

QCM-D Au-crystals coated with electrospun nanofibers (cross-linked and non cross-linked) were mounted in the QCM flow cell. After establishing a stable change in frequency and dissipation shift in air, MilliQ-water was introduced into the QCM chambers for 30–45 min. The flow rate of water and temperature were kept at 0.1 mL min^−1^ and 37 ± 0.1 °C, respectively, throughout all experiments.

### 2.9 *In vitro* drug release

*In vitro* drug release studies of DCF loaded electrospun nanofibrous substrates were performed using an Automated Transdermal Diffusion Cells Sampling System (Logan System 912-6, Somerset, USA). Drug loaded samples were cut into 1 cm × 1 cm and placed on the top of a sterilised polyethylene terephthalate (PET) mesh. Sterilization was performed using UV light with 30 minute exposure. The function of PET mesh was solely to assure constant wetting of the samples, as well as the prevention of their sinking into the Franz diffusion cell. The receptor compartment of the cells was filled with PBS (purchased from Sigma-Aldrich, Germany in the form of tablets) and its temperature was maintained at 37 °C. During the dissolution testing the medium was stirred continuously with a magnetic bar. Samples were collected over a period of 48 h at different time intervals, while the released/dissolved concentration of DCF in the receptor medium was determined by UV-Vis spectrophotometer (Cary 60 UV-Visible Spectrophotometer, Agilent, Germany) by quantification of the absorption bands at 276 nm (characteristic absorption maxima for DCF). The withdrawn sample volumes were replaced by fresh PBS. Due to sample withdrawal, followed by sample dilution through media replacement, sink conditions were assured. In calculation of concentrations using the Beer–Lambert law, this dilution was accounted for. All release studies were performed in three parallels.

The DCF loading content of the electrospun substrates were determined by the decrease of DCF concentration using UV-Vis spectroscopy against a standard curve at 276 nm.2
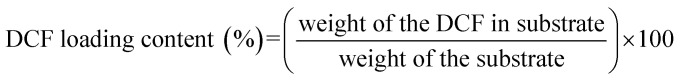


### 2.10 Biocompatibility studies

The electrospun samples were cut to dimensions of 1 cm × 1 cm and sterilised under the same procedure as described in Section 2.9. Advanced Dulbecco's Modified Eagle's Medium (DMEM) cell culture medium with 5 wt% fetal bovine serum (FBS) (Life technologies, Thermo Fisher Scientific Inc., Germany) was added to each sample and incubated for 24 h at 37 °C in 5 wt% CO_2_ atm. The samples (supernatants of the starting samples) were then added to a P96 microlitre plate with skin fibroblast cell culture (ATCC-CCL-110, Detroit 551, LGC Standards, UK). The following dilutions were used: 1 : 2, 1 : 4, 1 : 8, 1 : 16 and 1 : 32, each in 4 replicates. For the control, Advanced DMEM with 5 wt% FBS was used. The samples with cell cultures were incubated for another 24 h at 37 °C in 5 wt% CO_2_ atm. After 24 h the cells were stained with 0.1% crystal violet and the absorbance was measured at 595 nm. The obtained results were subjected to statistical analysis as described in Section 2.7.

## 3. Results and discussion

### 3.1 Conductivity, viscosity and surface tension

**Fig. 1:**
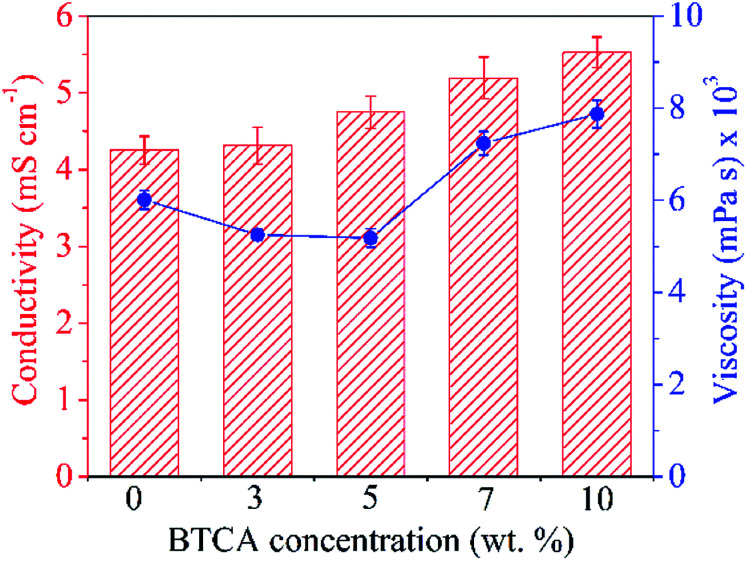
Conductivity (left) and viscosity (right) of electrospinning CMC/PEG solution added with different concentrations of BTCA.

It is well-established that the morphology and diameter of electrospun nanofibers can be substantially influenced by the properties of the electrospinning solution such as conductivity, viscosity and surface tension, *etc.*^34^ Therefore, prior to the formation of nanofibers, the said properties of CMC/PEG solution admixed with the cross-linker and catalyst (BTCA/SHP) at different concentrations (3, 5, 7 and 10 wt%) were investigated and the results are presented in Fig. 1. The addition of BTCA to CMC/PEG solution at all four concentrations does not change the surface tension and the observed values are close to *ca.* 60 mN m^−1^ (ESI, Table S1†). However, it does influence the conductivity and viscosity of solution. The measured conductivity of CMC/PEG solution (BTCA_0%) is due to the –COO^−^ groups of CMC. Further increase of conductivity of the solution with increasing BTCA concentration is because of the –COO^−^ groups of BTCA; as the latter possess four –COO^−^ groups in its structure. This phenomenon can efficiently prevent the formation of beads and increase the continuous spinning of uniform and small fibre diameter of CMC/PEG due to increased charge density on the surface of the ejected jet in electrospinning field.^35^

The influence of added BTCA on the viscosity of the CMC/PEG solution can be clearly seen in Fig. 1. It is expected that the addition of BTCA would lower the pH of CMC/PEG solution from neutral to acidic, leading to reduced electrostatic repulsion between the CMC chains, and thus increased viscosity. Although the measured pH of the solution is 2.5, the viscosity of the solution is slightly reduced after the addition of BTCA from 3 to 5 wt%. This can be explained, firstly, not all the –COO^−^ groups of CMC are protonated at lower concentration of BTCA and secondly, due to the weak interactions and entanglements between the chains of PEG, or CMC or CMC-PEG, which is caused by the added BTCA molecules. This might allow the polymer molecules to align much easier. However, at higher BTCA concentration (7–10 wt%), the viscosity of the solution is increased, which can be explained by reduced solubility, electrostatic repulsion between the CMC chains, and increased interactions between CMC and PEG chains.

### 3.2 Morphology, diameter and surface area of electrospun nanofibers

**Fig. 2:**
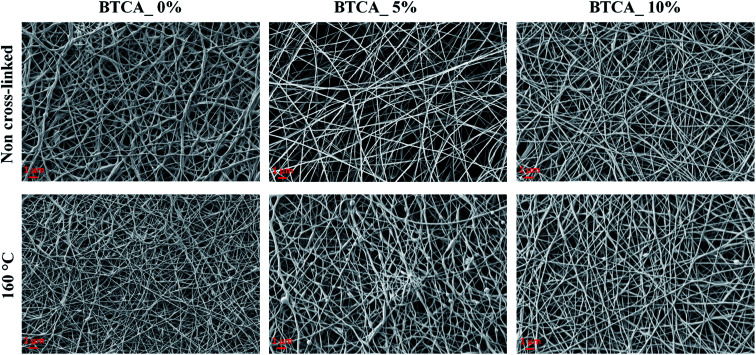
SEM morphology of non cross-linked and cross-linked electrospun nanofibers with different BTCA concentrations at 160 °C.

It is known that CMC, due to its carboxylic functional groups, increases the charge density of the solution in ejected jet. This, usually, causes strong elongation forces because of self-repulsion of excess charge density under electric field, leading to the formation of straight and regular nanofibers with thinner diameter.^36^ On the other hand, the uncharged polymer, PEG alone leads to the formation of fibers of thicker diameter, owing to its solution's unbalanced charging and jet stability under the electric field.^37^ Clearly, creating a balance between the surface tension of ejected jet and electrical force is crucial to create a regular and uniform electrospun nanofibers; which can be overcome by using the combination of CMC and PEG, as proven in our previous studies.^28,29^Fig. 2 shows the morphology of non cross-linked and cross-linked electrospun nanofibers with 0, 5 and 10 wt% BTCA at 160 °C. Results from other BTCA concentrations and temperatures, and drug incorporated samples are given in the supporting information (Fig. S1†). A beadless, long and continuous nanofibers are observed for BTCA-free and non-heated CMC/PEG (0 wt%) sample. After cross-linking with BTCA and incorporation of DCF (ESI, Fig. S2†), the electrospun nanofibers still kept their uniformity, morphology and structure. In addition, the presence of drug (ESI, Fig. S2†) and BTCA (at all concentrations) on the fiber's surface could not be seen, suggesting that the BTCA as well as drug molecules are homogeneously embedded into the CMC/PEG nanofibers. However, the BTCA-free CMC/PEG electrospun substrates are water soluble (see Fig. 6), which makes them unsuitable for long-term applications such as drug delivery and cell growth. To surmount this downside, BTCA was added to CMC/PEG solution and subjected to thermal cross-linking as stated above.

**Fig. 3:**
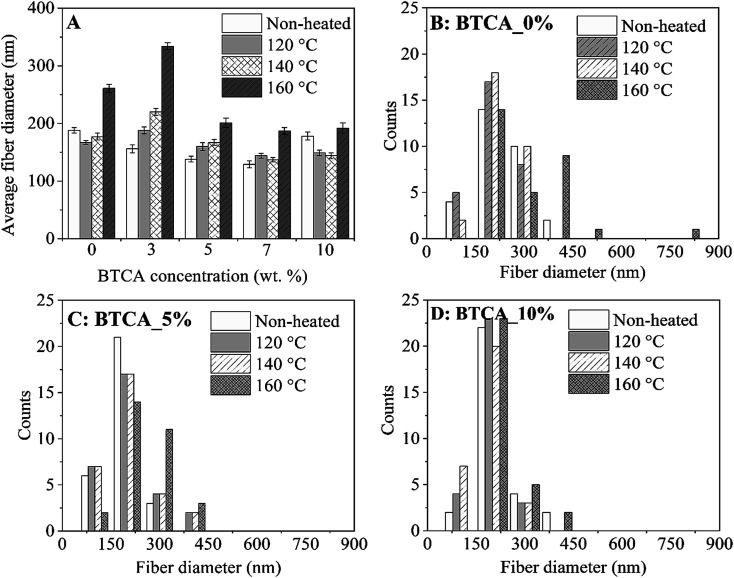
Average fiber diameter (A) and diameter distributions (B–D) of electrospun nanofibers cross-linked with different BTCA concentrations at different temperatures.

Since studies such as cell growth and drug release can be affected by fiber size, it is important to control the average diameter of fiber; the formation of it is a function of materials choice and electrospinning processing parameter, for example cross-linker concentration, viscosity, *etc.* The effect of BTCA concentrations and temperatures on the fiber's average diameter (Fig. 3A) and fiber distributions (Fig. 3B–D) can be easily noticed. Firstly, for non cross-linked samples (white bar, Fig. 3A), the average fiber diameter at BTCA_3% is slightly reduced compared to BTCA-free CMC/PEG electrospun nanofibers (diameter: 188 ± 4 nm) and then it increased to 178 ± 5 nm for BTCA_10%. This data fits well with the surface area results where the reduction of average fiber diameter resulted in increased specific surface area and *vice versa* (Table 1). It seems that electrospinning solution concentration, one of the critical parameters, plays a key role in controlling the fiber diameter. Since the concentration is directly related to viscosity (besides other parameters) of the electrospinning solution, it is suggested that the decreased viscosity and increased conductivity of the solution resulted in reduced average fiber diameter, improved fiber size, and formation of a thin and inter-connected fiber network/structures.^38^

**Table 1:** BET analysis of electrospun nanofibers before and after cross-linking with different concentrations of BTCA at 160 °C. Statistical significance is defined as *P < 0.05 compared to control sample (ANOVA test)

BTCA conc.	Surface area (m^2^ g^−1^)		Pore volume (cm^3^ g^−1^)		Pore size (nm)	
	Non cross-linked	160 °C	Non cross-linked	160 °C	Non cross-linked	160 °C
0	7.67 ± 0.2	3.08 ± 0.06	0.014 ± 0.001	0.006 ± 0.001	7.25 ± 0.2	8.08 ± 0.4
3	9.55 ± 0.3*	2.69 ± 0.1	0.017 ± 0.001	0.004 ± 0.002	7.21 ± 0.4	6.03 ± 0.8*
5	11.37 ± 0.1*	3.21 ± 0.1	0.019 ± 0.002*	0.005 ± 0.001	6.75 ± 0.2	5.59 ± 0.4
7	13.73 ± 0.4*	3.71 ± 0.06	0.023 ± 0.002*	0.005 ± 0.002	6.71 ± 0.3	5.79 ± 0.2
10	10.06 ± 0.3*	2.68 ± 0.04*	0.018 ± 0.001*	0.004 ± 0.001	7.09 ± 0.2	5.92 ± 0.3

Secondly, in general, with increasing temperature from 120 °C to 160 °C, the average fiber diameter is increased for each BTCA concentration except for BTCA_10%. Maximum average fiber diameter (334 ± 6 nm) is obtained for BTCA_3% at 160 °C comparing to non-heated sample of the same concentration, and BTCA-free (0 wt%) sample. It is obvious that cross-linking with BTCA and increase of temperature lead to increased average fiber diameter, and also affected the distribution of fiber diameters (Fig. 3B–D). This can be attributed to the conformational changes of the polymer chains, which are then linked together by ester-bonds, leading to nanofibers with larger diameters. After cross-linking with BTCA_3%, the average fiber diameter of electrospun sample does not increase substantially regardless of the temperatures. They, for example, remained almost constant (*ca.* 195 nm) at 160 °C for each BTCA concentration. The BET analysis performed for electrospun nanofibers before and after thermal cross-linking with BTCA_3–10% at 160 °C is shown in Table 1. The statistical analysis showed that the surface area and pore volume of non-cross-linked samples is significantly different at higher BTCA concentration (5–7 wt%) compared to BTCA_0% (control sample). Whereas in the case of pore size no significant differences are found for non cross-linked samples. Even though the reduction of surface area, average pore volume and pore size are obvious after cross-linking with BTCA, the observed values of BET parameters (surface area to pore size) are not statistically different when the BTCA concentration is increased from 3 to 10 wt% than that of control sample. The reduction of pore size and pore volume for cross-linked samples can be explained that the junctions between the nanofibers brought closer by the ester-bond linkage and the fiber mat is denser as the result of cross-linking. This behavior is consistent with the previously reported papers.^39,40^

### 3.3 Infrared analysis

**Fig. 4:**
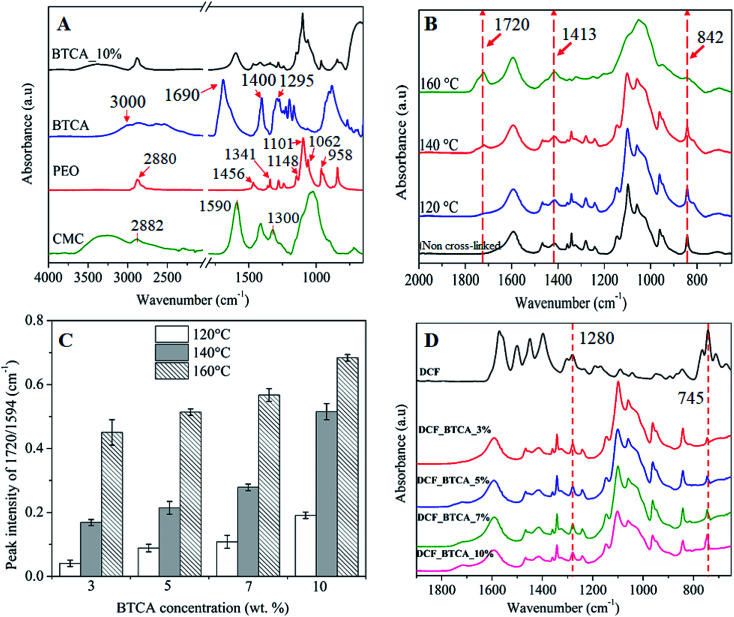
(A) ATR-FTIR absorption spectra of neat PEG, Na-CMC and BTCA, and non-heated CMC/PEG nanofibers with added BTCA_10%. (B) Spectrum of electrospun nanofibers cross-linked with BTCA_10% at different temperatures, (C) the absorption peak intensity ratio (1720 cm^−1^/1594 cm^−1^) of electrospun samples cross-linked with different BTCA concentrations and temperatures, (D) spectrum of pure DCF and DCF incorporated electrospun nanofibers cross-linked with different BTCA concentrations at 160 °C.

Fig. 4A shows the ATR-FTIR spectra of neat CMC, PEG, BTCA, and non-heated CMC/PEG electrospun sample containing BTCA_10% (as described in Section 2.2). The characteristic peaks of CMC are observed at 3200–3600 cm^−1^ (OH stretching), 2882 cm^−1^ (C–H stretching vibration), 1590 cm^−1^ (–C=O, carbonyl stretching of carboxyl group) and at1300 cm^−1^ (–CH_2_ stretching), respectively. The peaks that are centered between 1000 and 1200 cm^−1^ are attributed to –C–OH stretching vibrations and the C–O–C glycosidic bond of CMC.^26,41^ All characteristic peaks of PEG are observed at 1148, 1101, 1062, and 958 cm^−1^ (C–O–C stretching vibration), 1456 cm^−1^ (–CH_2_ scissoring), 1341 cm^−1^ (–CH_2_ deformation mode), 1240 cm^−1^ (–C–O stretching), and at 2880 cm^−1^ (C–H symmetric stretching),^42^ respectively. BTCA gives a sharp absorption peak at 1690 cm^−1^, corresponding to –C=O group of the carboxylic acid groups. In addition, the broad peak and sharp peak centered at 3000 cm^−1^ and 1400 cm^−1^ are due to –OH and –CH_2_ stretching mode. The other two peaks centered at 1295 cm^−1^ and 933 cm^−1^ are associated to –C–O stretching and –OH out-of-plan deformation of H-bonded carboxylic groups.^43^ Although all characteristic peaks of neat polymer are observed for non-heated CMC/PEG nanofibers added with BTCA_10%, the –C=O peak at 1690 cm^−1^ from the contribution of BTCA is absent. This suggest that the cross-linker BTCA is well-shielded by the polymeric networks of CMC and PEG.

ATR-FTIR spectra of electrospun nanofibers cross-linked with BTCA_10% at different temperatures (120–160 °C) is shown in Fig. 4B. Comparing the mentioned spectra before and after cross-linking, the emergence of several new peaks is obvious. The most noticeable change occurred in the region between 1500 cm^−1^ and 1800 cm^−1^ for the cross-linked samples. Along with the carboxyl carbonyl peak at 1590 cm^−1^, a new peak arises at 1720 cm^−1^, which is attributed to ester carbonyl peak. The latter peak is absent for both non-heated BTCA_10% and BTCA_0% samples (data not shown). Similar phenomenon is also noticed for other BTCA_*x* (*x* = 3, 5 and 7 wt%, ESI, Fig. S3†). The observed new peak at 1720 cm^−1^ confirms that the carboxylic acid groups of BTCA are cross-linked to hydroxyl groups of CMC and PEG *via* ester bonds. Comparing other reported works that employed toxic chemical agents for cross-linking and stabilization of nanofibers,^27^ the reported approach in this work possess certain advantages in terms of green nature, simplicity, and non-toxicity, respectively.

Interestingly, the intensity of peak at 1720 cm^−1^ is increased with the raise in temperature from 120 °C to 160 °C, an indication that more number of ester-bonds or ester-linkages are created between BTCA and CMC/PEG. As a result, we evaluated the ester cross-linkages semi-quantitatively by measuring the ester carbonyl and carboxyl carbonyl peak intensity ratio (1720 cm^−1^/1590 cm^−1^).^44^ As shown in Fig. 4C, the ratios of the mentioned peaks intensity increased not only with increasing concentrations of BTCA but also with increased temperature, demonstrating that increasing of both parameters (concentration of BTCA and temperature) favors the esterification process. The intensity ratio is two-fold higher at 160 °C for each BTCA concentration when comparing to samples cross-linked at 120 °C. Results from these semi-quantitative data confirms that both CMC and PEG can be esterified or cross-linked easily and efficiently either by increasing of BTCA concentration or temperatures.

Also, there are changes in the absorption pattern of glucopyranose ring of CMC *i.e.* in the region 800–1450 cm^−1^ as can be seen in Fig. 4B. The observed peak at 1413 cm^−1^ (–CH_2_ bending vibration) and 842 cm^−1^ (C–O–C stretching at β-(1→4) glycosidic bonds) is associated with the crystalline and amorphous structure of cellulose, as reported by several others.^45,46^ The absorption peak intensity at 1413 cm^−1^ is increased while it is decreased substantially for the peak at 842 cm^−1^ with increasing temperatures. This behavior is more pronounced, especially, at 160 °C, where the amorphous peak (842 cm^−1^) is almost disappearing. A most likely scenario is that a rearrangement of the amorphous and crystalline regions in the CMC nanofibers structure occurred, leading to increased crystalline domains with concomitant decrease of amorphous domains upon exposing the CMC to higher temperatures.

ATR-FTIR spectrum of DCF incorporated samples is shown in Fig. 4D. For pure DCF, all characteristic absorption peaks are observed at 3253 cm^−1^ (–NH stretching of the secondary amine, supporting information, Fig. S3†), 1571 cm^−1^ (–C=O stretching of the carboxyl ion), 1500 cm^−1^ (–C=C aromatic ring stretching), 1450 cm^−1^ (–CH_2_ bending), at 1280 and 945 cm^−1^ (–C–O–C stretching), and at 745 cm^−1^ (–C–Cl stretching).^47,48^ All samples cross-linked with different concentrations of BTCA showed the –C–O–C and –C–Cl stretching peaks of DCF at 1280 cm^−1^ and 745 cm^−1^, respectively, indicating that DCF is successfully incorporated into the nanofibers.^49^ Also, the characteristic peaks of CMC and PEG are not shifted, implying the absence of any chemical interactions of DCF with polymer functional groups, meaning that the drug molecules are physically incorporated into the nanofibrous matrices. The physical incorporation of DCF has certain advantageous that it can be diffused and released into the medium without any hinderance from the polymer matrix.

### 3.4 Wettability, swelling and stability of electrospun nanofibrous substrates

**Fig. 5:**
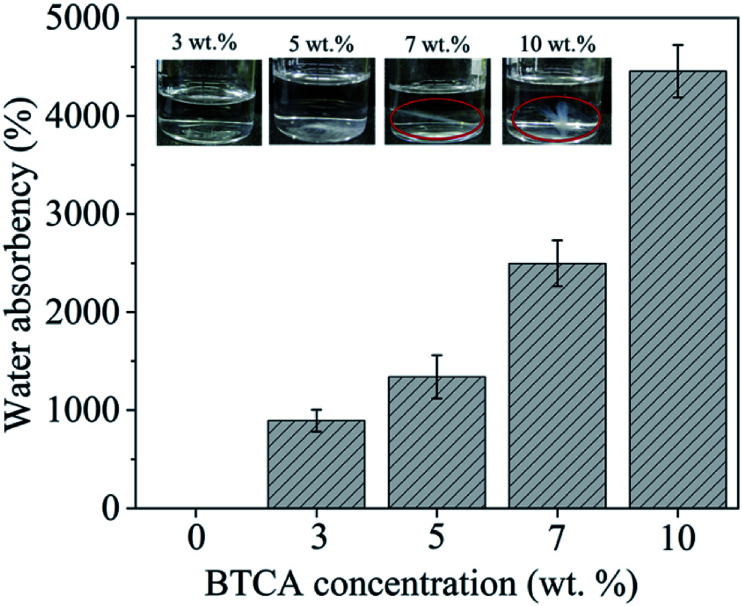
Water absorption capacity (WAC) and photo images of electrospun samples cross-linked with different BTCA concentrations at 160 °C, after 24 h soaking in water.

The wettability, water absorption capacity (WAC) and dimensional stability of electrospun nanofibrous substrates cross-linked with BTCA_*x* (*x* = 0, 3, 5, 7, 10 wt%) at 160 °C were investigated. The static water contact angle SCA(H_2_O) of CMC/PEG nanofibrous substrate cross-linked with 0 and 10 wt% BTCA at 160 °C was not possible to measure as they are too hydrophilic; as confirmed by the rapid absorption (*ca.* 1 s) of water droplet when placed it on the substrate (see ESI, Fig. S4†). The WAC of BTCA-free (0 wt%) nanofibrous substrate, heated at 160 °C, is not given in Fig. 5 since they are dissolved rapidly and completely when immersed in aqueous swelling medium. Interestingly, the WAC of nanofibrous substrates is increased with increasing BTCA concentration, but it is more pronounced for cross-linked (BTCA_7–10%) samples. This seems that the nanofibers (substrates) become more and more hydrophilic after BTCA addition and cross-linking. According to Basu *et al.* the addition of PEG to CMC does not increase WAC at neutral pH.^50^ This leads to the conclusion that the deprotonated COO^−^ groups of CMC and BTCA, at neutral pH, contribute mainly to the water uptake. Besides to their high WAC (BTCA_7%: 2500%, BTCA_10%: 5500%) and hydrophilicity, the nanofibrous substrates are stable and are not dissolved in water even after 48 h (see the photo images, Fig. 5 inset). At lower BTCA concentrations (3–5 wt%), no remainders of the nanofibrous substrates or any visible residues could be seen already after 28 h, an indication that the cross-linking with lower amounts of BTCA (3–5 wt%) are inefficient, leading to a complete dissolution of CMC/PEG polymer chains. This is also in good agreement with IR results where the maximum ester carbonyl bonds are formed at higher BTCA concentrations (7–10 wt%) at 160 °C (Fig. 4C).

**Fig. 6:**
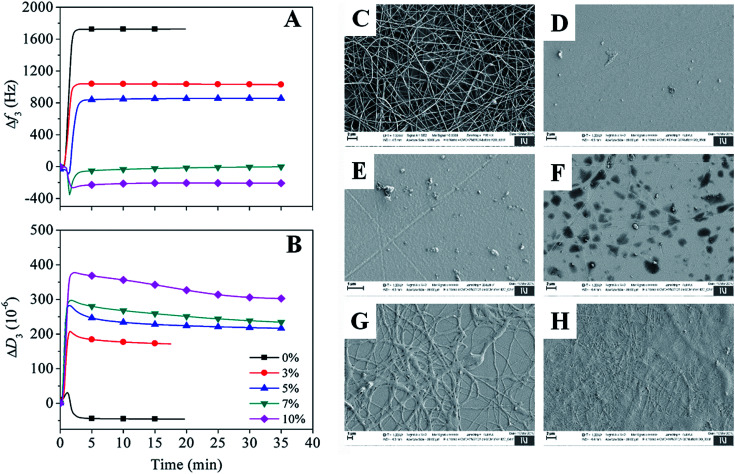
(A and B) QCM-D change in frequency and dissipation shifts of electrospun samples cross-linked with different BTCA concentrations at 160 °C upon exposure to water. Electrospun nanofibers of BTCA_0% on QCM-D crystals before (C) and after (D) rinsing with water, (E) BTCA_3%, (F) BTCA_5%, (G) BTCA_7% and (H) BTCA_10% cross-linked electrospun nanofibers at 160 °C, after interaction with water.

In addition to the above conventional approach, a highly sensitive and complementary technique *i.e.*, QCM-D, which is often employed to study water interaction capacity of supported nano-sized cellulose films,^51,52^ was used to analyse the swelling and stability of the electrospun nanofibers. In Fig. 6, the QCM-D change in frequency (A) and dissipation (B) for the electrospun nanofibers, cross-linked with different BTCA concentrations at 160 °C, upon interaction with water is shown. For BTCA-free sample (0 wt%), as soon as the water interacts with the sample surface a steep increase of frequency shift for the first few minutes followed by a steady state (no change in frequency shift) already before 5 min is observable. The shift in dissipation, used to measure the rigidity of the adsorbed layer, in particular, is increased initially and then decreased to negative value (Δ*D*_3_: −44 × 10^−6^) followed by the steady state. An increase of frequency shift and decrease of dissipation shift upon contact with water indicates the removal of loosely attached nanofibers to the QCM crystals or the dissolution of polymer nanofibers or the combination of both. This is also reflected for samples cross-linked with lower BTCA concentrations (3–5 wt% BTCA), where increased frequency shift is observed, but lower than the value observed for BTCA-free (0 wt%) sample. The increased dissipation shift can be related to the swelling of undissolved polymers, which remained on the crystal surface. These findings can be further confirmed from the SEM results. The latter shows the formation of a thin layer of nanofibers from BTCA-free sample (C), which is due to a shorter time (10 s), used for electrospinning of the solutions. This was done because the coating of nanofibers on the QCM Au-crystals should be thin enough to guarantee proper sensitivity of the QCM-D. After exposure to water, the cross-linked nanofibers are not visible anymore on the crystal surface for 3 wt% (E) and 5 wt% (F) BTCA as that of BTCA-free (0 wt%) non cross-linked electrospun samples (D). This confirms that the applied 3 to 5 wt% BTCA is still too low to create sufficient ester-linkages and form water insoluble CMC/PEG nanofibers, as also noticed in the case of swelling and stability analysis (Fig. 5). Interestingly, for the samples cross-linked with higher BTCA concentrations, the negative frequency shift (7 wt%: −9 ± 2 Hz, 10 wt%: −209 ± 6 Hz) is observed while the increase of dissipation shift is positive when they were brought into contact with water. The observed negative frequency shift indicating that the nanofibers are not dissolved and firmly attached to the crystal surface. This is further proven by SEM data where the nanofibers are still visible on the crystal surface in the case of BTCA_7% (G), and even more are present for BTCA_10% (H). Not like in the case of lower BTCA concentrations, the nanofibers are covalently and tightly attached together by maximum number ester-linkages at the highest BTCA concentration (7–10 wt%), leading to water insoluble materials. As the BTCA concentration is increased from 7 to 10 wt%, the negative frequency shift and positive dissipation shift is also increased, indicating that the swelling capacity of the nanofiber layer is increased, and thus the layer becomes increasingly viscoelastic.

### 3.5 *In vitro* drug release studies

**Fig. 7:**
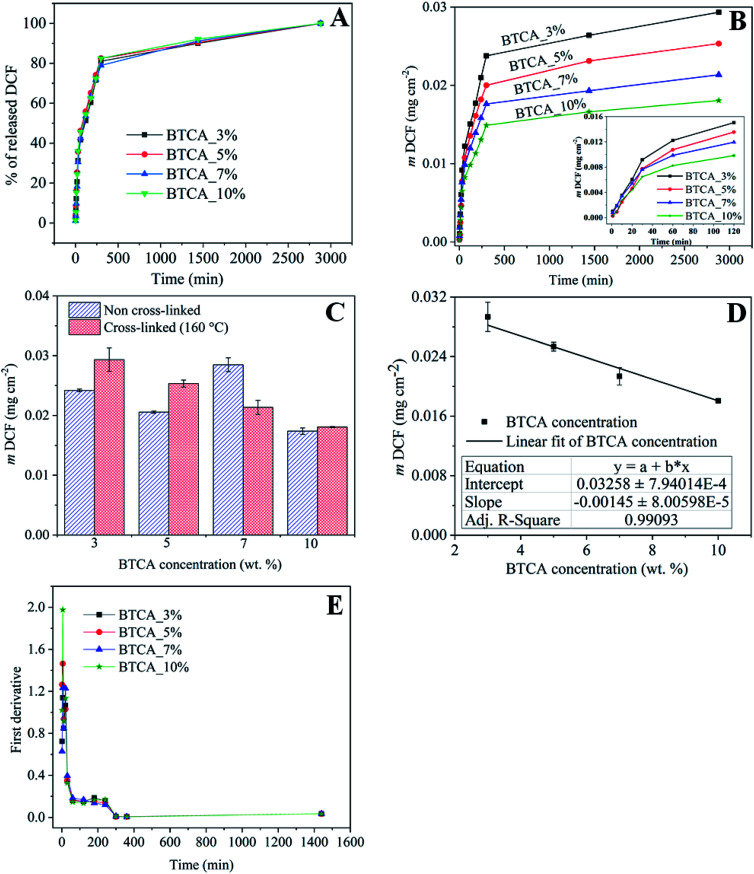
(A) DCF mass as a function of time, (B) the % release of DCF as a function from electrospun samples cross-linked with different BTCA concentrations at 160 °C, (C) total amount of drug release from the non cross-linked and cross-linked (at 160 °C) electrospun nanofibers with different BTCA concentrations, (D) linear fitting of the overall drug release from the cross-linked (at 160 °C) nanofibrous substrates with BTCA as in Fig. 8B. (E) First derivatives of the release data from (A).

Ideally, drug loaded wound healing materials should facilitate sustained drug delivery (for the time until wound dressing exchange) and maintain minimal drug-skin contact to overcome local irritation, itching and allergies. Therefore, the use of ECM resembling nanofibrous substrates, which are in three-dimensional (3D) form and stable at physiological environments, are beneficial because of its capacity to effectively control diffusion and sustain the release of NSAID therapeutic molecules. The *in vitro* release of DCF in percentage (%) and cumulative amount (mg cm^−2^) as a function of time in PBS at pH 7.4 from nanofibrous samples cross-linked with different BTCA concentrations at 160 °C are shown in Fig. 7A and B. To verify if there is any interference of CMC/PEO nanofibers in the absorbance of DCF incorporated samples, the UV-Vis spectra of BTCA_10% (drug free) and DCF_BTCA_10% were recorded for comparison and the results are shown in ESI (Fig. S5†). The presence of no peak at 276 nm for BTCA_10% sample suggest that their interference in the DCF incorporated sample can be neglected. The drug loading content for both non cross-linked and all cross-linked samples were determined to be in the range of *ca.* 30%. Even though the release pattern of DCF is similar for all cross-linked samples from varying concentrations of BTCA, the DCF is released in several steps for each BTCA concentration. Release of DCF is due to the diffusion of water into the nanofibrous matrix, leading to swelling of the polymer, as demonstrated by swelling studies (Fig. 5 and 6). Swelling of the polymer enabled the encapsulated drug to diffuse out and be released into surrounding media. In this context, highly swollen matrices should release higher amounts of the drug. All samples burst in the initial stages (first few minutes) of drug release, which can be caused by the adsorbed DCF on the surface and pores of nanofibers during the electrospinning process.^53^ Even though the differences in the overall released mass of DCF (Fig. 7B) is small during 5–30 min (burst period), they continued to increase for each BTCA concentration till the end of the measurements (48 h). For example, the release mass of DCF is approximately 6% (0.0015 mg cm^−2^) for the first 5 min. After 2 h, the calculated released masses of DCF are in the following order: 0.01505 (BTCA_3%) > 0.01355 (BTCA_5%) > 0.01196 (BTCA_7%) > 0.00983 (BTCA_10%). The released mass at this point is in the range of *ca.* 50% for each BTCA concentration. All cross-linked systems continue to release DCF and no steady-state is reached within 48 h. But the percentage release profile (Fig. 7A) showed that all incorporated DCF is released from all samples within this time frame, which corresponds to the time until most of the wound dressings are exchanged in clinical wound care. The overall released mass of DCF from the non cross-linked and cross-linked samples with different concentrations of BTCA at 160 °C is depicted in Fig. 7C. It is obvious that the overall release rate (Fig. 7B) and mass (Fig. 7C–D) is higher at 3% BTCA (*m*DCF: 0.029 ± 0.002 mg cm^−2^) and slow down with increasing concentration of BTCA. The latter, also, resulted in the linear reduction of release rate/mass with a correlation coefficient of 0.99 (Fig. 7D). It is expected that the strongly swollen and hydrated CMC/PEG nanofibrous substates, which are cross-linked with higher concentration of BTCA (7–10 wt%, see Fig. 6), would lead to more released mass of DCF. However, an opposite behavior is noticed as shown in Fig. 7B. The reduction of overall released mass as a function of BTCA concentrations can be related to the porous structure of nanofibrous substrates. It is suggested that increased specific surface area and smaller pore size (Table 1), in addition to the increased dense network structure, limited the dissolution and diffusion of DCF through cross-linked nanofibrous network of CMC/PEG.

In addition, the cross-linking density of BTCA can influence the release rate of DCF. It is proposed that the low cross-linking density at lower BTCA concentrations, as confirmed by ATR-FTIR results (Fig. 4C), facilitated maximum diffusion, and thus leading to quick and higher released DCF mass. This can be further supported by swelling studies where the samples cross-linked with 3–5 wt% BTCA dissolved rapidly upon contact with water (Fig. 5). In contrast, the electrospun samples with high cross-linking density, achieved using 7–10 wt% BTCA, maintained the morphology and structural integrity/network of the nanofibrous substrates during the entire drug release period, allowing limited diffusion and release of DCF. This also means that the swollen 3D network of nanofibrous substrates is not collapsed or dissolved (like in the case of BTCA_3% and BTCA_5%) upon contact with release medium. QCM-D stability studies and SEM results also confirmed that the morphology and 3D network of electrospun samples cross-linked samples with 7–10 wt% BTCA are retained when they are brought into contact with water. Overall, these results lead to the conclusion that the release rate or kinetics can be well-controlled by tuning the cross-linker concentration. On the other hand, while no linear trend can be found for the non cross-linked samples, the overall released mass is lower for 3–5 wt% BTCA, higher at 7% BTCA, and become comparable with 10% BTCA cross-linked samples (Fig. 7C). In addition to stability issue, the handling of non cross-linked electrospun nanofibrous substrates is difficult especially in release or cell culturing medium. Therefore, no further studies have been carried out for non cross-linked samples.

**Fig. 8:**
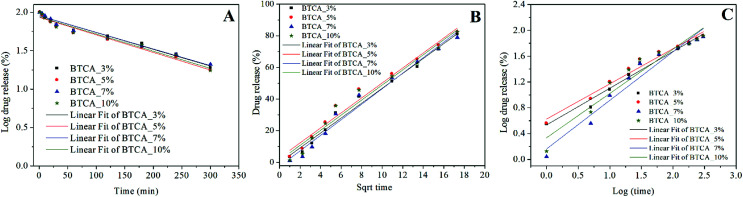
Kinetics models fitted to the release data of DCF, incorporated electrospun samples cross-linked with different BTCA concentrations at 160 °C. (A) First-order, (B) Higuchi-model, (C) Korsmeyer-peppas model.

The release data was further evaluated and fitted by linear regression analysis according to three different kinetic models to understand the release mechanism of DCF from BTCA cross-linked CMC/PEG nanofibrous substrates. The kinetic models such as the first-order (eqn (3)), Higuchi-model (eqn (4)) and Korsmeyer–Peppas model (eqn (5)) gave the best fit and the highest square of correlation coefficient (*r*^2^) value for the release data out of the five kinetics models, which were considered for the data fitting. The latter was done for the release data of DCF from 0–300 min. When compared with the other above mentioned two kinetic models, the first order model resulted in the lowest *r*^2^ value, and therefore not considered to explain the release kinetics of DCF. As can be seen in Fig. 8 B–C and Table 2, the fitting results show that the release kinetics fit is more consistent with Higuchi model following the Korsmeyer–Peppas model. While the Higuchi model gave the best fit and the highest *r*^2^ value (0.966–0.985) for all BTCA cross-linked samples, the Korsmeyer–Peppas model showed the best fit and the highest *r*^2^ value (0.973–0.976) only up to 5 wt% BTCA cross-linked samples. Moreover, the calculated *r*^2^ value is slightly lower for the latter model. Overall, the obtained best fits and the highest correlation coefficient values for both models indicate that the fitting results can be correlated well with the real relationship between the response data parameters.

**Table 2:** The correlation coefficient (*r*^2^) and release component (*n*) calculated from different kinetic models for DCF loaded system

BTCA conc. (wt%)	First order	Higuchi model	Korsmeyer-Peppas model	
	*r* ^2^	*r* ^2^	*r* ^2^	*n*
3	0.975	0.985	0.976	0.534
5	0.969	0.976	0.973	0.626
7	0.973	0.978	0.954	0.163
10	0.961	0.966	0.928	0.336

In general, the First-order kinetics is employed to relate the drug release where the release kinetics are concentration depended.

3 log *Q* = log *Q*_0_ − *K*_*t*_/2.303

where *Q* is the amount of released drug in time ‘*t*’, *Q*_0_ is the initial concentration of the drug and *K* is the first-order rate constant.

The Higuchi model uses pseudo-steady-state assumptions to describe the release kinetics of drug from several types of pharmaceutical forms (*e.g.*, porous matrix) based on Fick's law, showing a square root of time dependent process.^54^

4 *Q* = *Kt*^1/2^

where *Q* is the amount of drug released at time ‘*t*’ and *K* is the Higuchi constant.

On the other hand, the Korsmeyer–Peppas model, derived from Fick's law, is mainly used to describe the release kinetics of drug from polymeric. This model can be defined as^54^

5 *Q*_*t*_/*Q*_∝_ = *Kt*^*n*^

Where *Q*_*t*_ and Q_∝_ is the amount of drug released in time *t* and released after infinitive time, *K* is the kinetic constant and the exponent ‘*n*’ is the release characteristic index, which is used to characterize the release mechanism. If the exponent ‘*n*’ is less than 0.45, then the release mechanism is Fick diffusion and it is primarily based on drug diffusion. The drug release and the release mechanism are non-Fick diffusion control when 0.45 < *n* < 0.89. When *n* > 0.89, the drug release is dominated by the combination of both diffusion and erosion-controlled process.

For cross-linked samples with 3–5 wt% BTCA, the highest *r*^2^ value is obtained with Higuchi model, confirming the *t*^1/2^ dependence of the drug release, which is purely the characteristic of Fickian diffusion mechanism. This means that the drug release is predominantly controlled by diffusion. On the other hand, the probability of drug release also *via* non-Fickian diffusion cannot be ruled out since the nanofibrous substrates cross-linked with lower BTCA concentration (3–5 wt%) were degrading within the time period of drug release. This can lead to release of drug simultaneously and rapidly from both the surface and bulk part of matrix, meaning that as the nanofiber is degraded or eroded, the drug is released from the entire volume of the matrix. In such a case, the drug is free to be released through diffusion mechanisms as well, in addition to the erosion controlled, as it is supported by the diffusional exponent ‘*n*’ values derived from the Korsmeyer–Peppas model, which are above 0.45. Also, the several breaks observed in the first derivatives profiles (Fig. 7D) derived from the release data depicts that multiple mechanisms are involved in the course of DCF release. This means that release of DCF from the hydrophilic electrospun nanofibers is caused not only by a “simple” diffusion-controlled mechanism but is often additionally accompanied by a combination of swelling followed by erosion of polymer chains when come into contact with a buffer solution. For 7 and 10 wt% BTCA cross-linked samples, which are stable and highly swollen, the release of drug is clearly the Fickian *i.e.* diffusion-controlled type as it is revealed from the highest *r*^2^ value according to the Higuchi model and from the exponent ‘*n* < 0.45’ value.

### 3.6 Biocompatibility studies

**Fig. 9:**
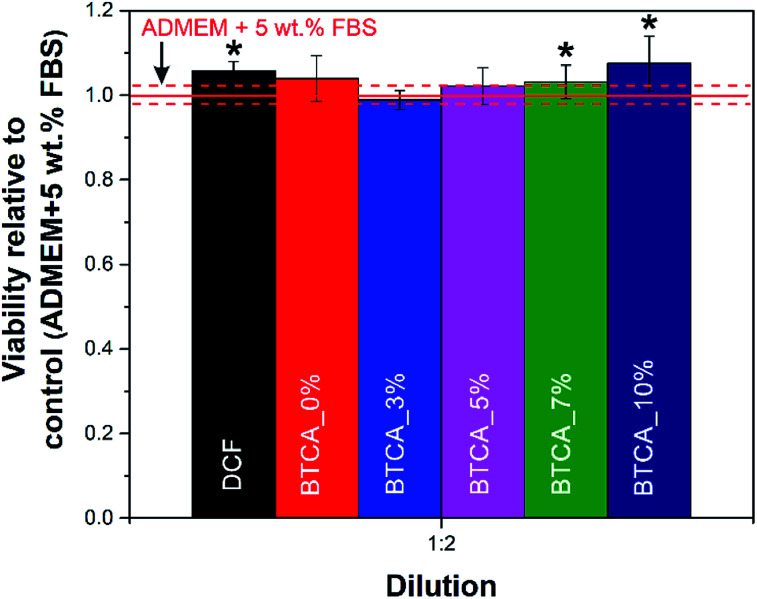
Viability of human skin derived fibroblasts after exposure to the DCF incorporated electrospun nanofibrous substrates cross-linked with different concentrations of BTCA at 160 °C. The shown results were calculated relative to control (pure cell growth media). The dashed red lines correspond to the calculated confidence intervals for the control sample. Values are expressed as percentage of the means ± SD. Statistical significance was defined as *P < 0.05 compared to control sample (ANOVA test).

The final set of experiments in this study was related to the testing of the prepared nanofibrous substrates potential to be used in biomedical applications, especially in treatment of wounds or skin tissue repair. For this purpose, we tested the influence of cross-linked nanofibers with different BTCA concentrations on the human skin derived fibroblast viability (based on crystal violet dyeing). Of course, the same test was initially used to evaluate the biocompatibility of the materials, but we immediately saw that these pose no harm to the used cells, but even promoted cell growth. The initial set of experiments was performed using the standard procedure to dilute the initial samples using the binary system (1 : 2, 1 : 4, 1 : 8,…), but since the sample have proven biocompatible already or the lowest of the dilutions (1 : 2), only these are included here for discussion (Fig. 9). All other results are shown in the supporting information (see Fig. S6†). From Fig. 9 it can be immediately observed that the viability of cells is significantly improved when exposed to pure DCF compared to control sample (pure cell growth media – ADMEM + 5 wt% FBS). Similarly, the DCF incorporated BTCA_7% and BTCA_10% samples, which were stable water (Fig. 5 and 6), showed significant differences in the viability in comparison with control and other CMC/PEG samples. Since it is known that cells “prosper” in more stable environments (with the smallest possible material degradation during their growth/exposure),^55^ the observed higher viabilities in the more cross-linked (highly stable) samples, seems logical. It is proposed that the highly cross-linked nanofibrous materials with BTCA_7% and BTCA_10% are less prone to degrade in the cell growth media than those of samples cross-linked with 0 to 5 wt% BTCA (that degraded or dissolved in water within 28 h). Obviously, the less degradation products, the higher the viability, which indirectly suggests that the degradation products of the prepared electrospun materials negatively affects the cell viability (although still no toxic effects were observed for neither of the samples). Based on the same amount (30%) of incorporated drugs in all samples, it is assumed that the drugs in the nanofibers did not significantly influence the cell viability as that of BTCA at higher concentration. Finally, all the results prove the suitability of the prepared materials to be further tested in relation to their potential application in either dermal wound healing or in skin tissue engineering applications in general.

## 4. Conclusions

Needles electrospinning technique was used for the fabrication of diclofenac (DCF) incorporated and stable nanofibrous substrates from the aqueous solution containing carboxymethyl cellulose (CMC), polyethylene glycol (PEG), and 1,2,3,4-butanetetracaboxylic acid (BTCA) respectively. Increasing, especially higher, concentrations of BTCA and temperatures favored the formation of ester-bonds and cross-linking of nanofibers, since the BTCA is activated more in the presence of sodium hypophosphite monohydrate catalyst at higher temperature. The thermally cross-linked samples showed decreased average fiber diameters, pore volumes and pore sizes, whereas the specific surface area of the nanofibers are increased. The stability and swelling capacity of the cross-linked nanofibrous substrates increased with increasing BTCA concentrations as proven by swelling and quartz crystal microbalance studies. Although a fast (burst) release followed by a diffusion controlled release of DCF are observed, the release kinetics are of the same type for all cross-linked nanofibrous samples. A linear decrease in the overall released mass is observed during the period of release time with increasing BTCA concentration, although the opposite behavior is anticipated. Results from the release data fitted using three kinetic models showed that the release of DCF follows the non-Fickian diffusion mechanism *i.e.* both diffusion and erosion controlled process at the lowest BTCA concentration based on the exponent ‘*n* < 0.45’ value derived from the Korsmeyer–Peppas model, although the Higuchi model gave the highest correlation coefficient *r*^2^ value. At the highest BTCA concentration, a clear diffusion control (Fickian mechanism) is observed according to the calculated ‘*n*’ value which is lower than 0.45. Even though no proliferation of fibroblast cells is induced, neither the polymers, cross-linker and nor the high concentration of drug decreased the viability of cells, a positive sign that the applied components are biocompatible. Overall the obtained results demonstrated that the developed stable electrospun nanofibrous system should have high advantages for the effective incorporation of all types of hydrophilic therapeutic molecules and give them a prolonged and controlled release for at least two days, so that the entire system can be even applied to treat chronic wounds, and in regenerative medicine in general.

## Conflicts of interest

There are no conflicts to declare.

## Supplementary Material

RA-009-C9RA03399H-s001
