# Supplementary material for: A green approach to obtain stable and hydrophilic cellulose-based electrospun nanofibrous substrates for sustained release of therapeutic molecules
Source: RSC Adv. 2019 Jul 9;9(37):21288–301. doi: 10.1039/c9ra03399h (PMC9066020; doi:10.1039/c9ra03399h)
Supplement: RA-009-C9RA03399H-s001 [file RA-009-C9RA03399H-s001.pdf]

Electronic Supplementary Material (ESI) for RSC Advances.
This journal is © The Royal Society of Chemistry 2019

Supporting information

A Green Approach to Obtain Stable and Hydrophilic Cellulose-based Electrospun Nanofibrous substrates for Sustained Release of Therapeutic Molecules

Manja Kurečič[a,b*], Tamilselvan Mohan[a,*], Natalija Virant[a], Uroš Maver[c], Janja Stergar[c], Lidija Gradišnik[c], Karin Stana Kleinschek[a], Silvo Hribernik[a,b]

†Laboratory for Characterization and Processing of Polymers, Faculty of Mechanical Engineering, University of Maribor, Smetanova 17, 2000 Maribor, Slovenia

$Faculty of Electrical Engineering and Computer Science, University of Maribor, Koroška cesta 46, SI-2000 Maribor, Slovenia

‡Institute of Biomedical Sciences, Faculty of Medicine, University of Maribor, Taborska ulica 8, 2000 Maribor, Slovenia

Table S1. Surface tension of CMC/PEG electrospinning solution added with different concentrations of BTCA.

BTCA concentration (%, w/w)	Surface tension (mN/m)
0	60.1
3	61.3
5	60.9
7	60.9
10	61.5

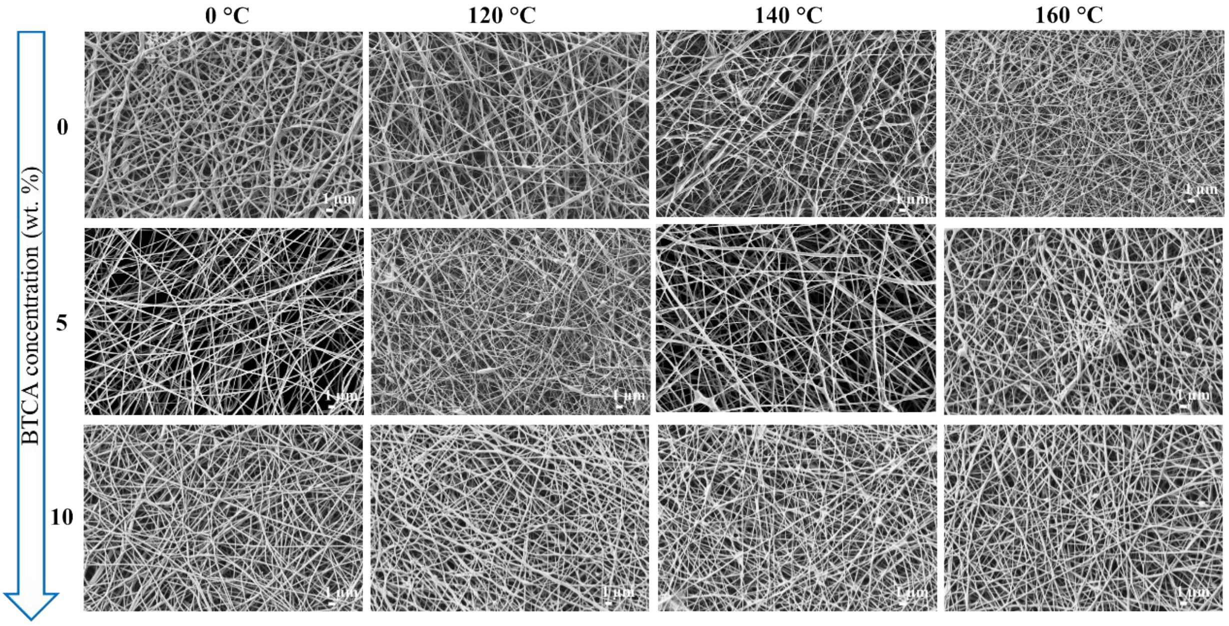

Fig. S1 SEM morphology of non cross-linked and cross-linked electrospun nanofibers with different BTCA concentrations at different temperatures.

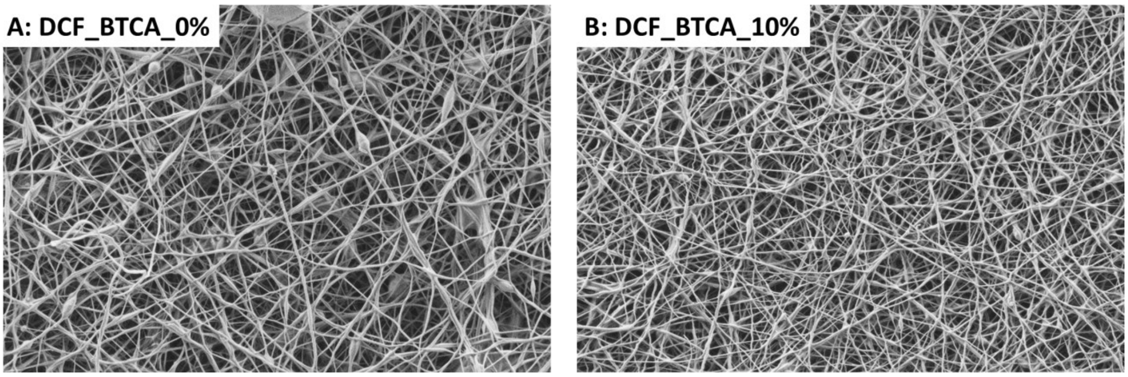

Fig. S2 SEM morphology of DCF incorporated and cross-linked with 0 wt. % (A) and 10 wt.% BTCA (B) at 160 °C.

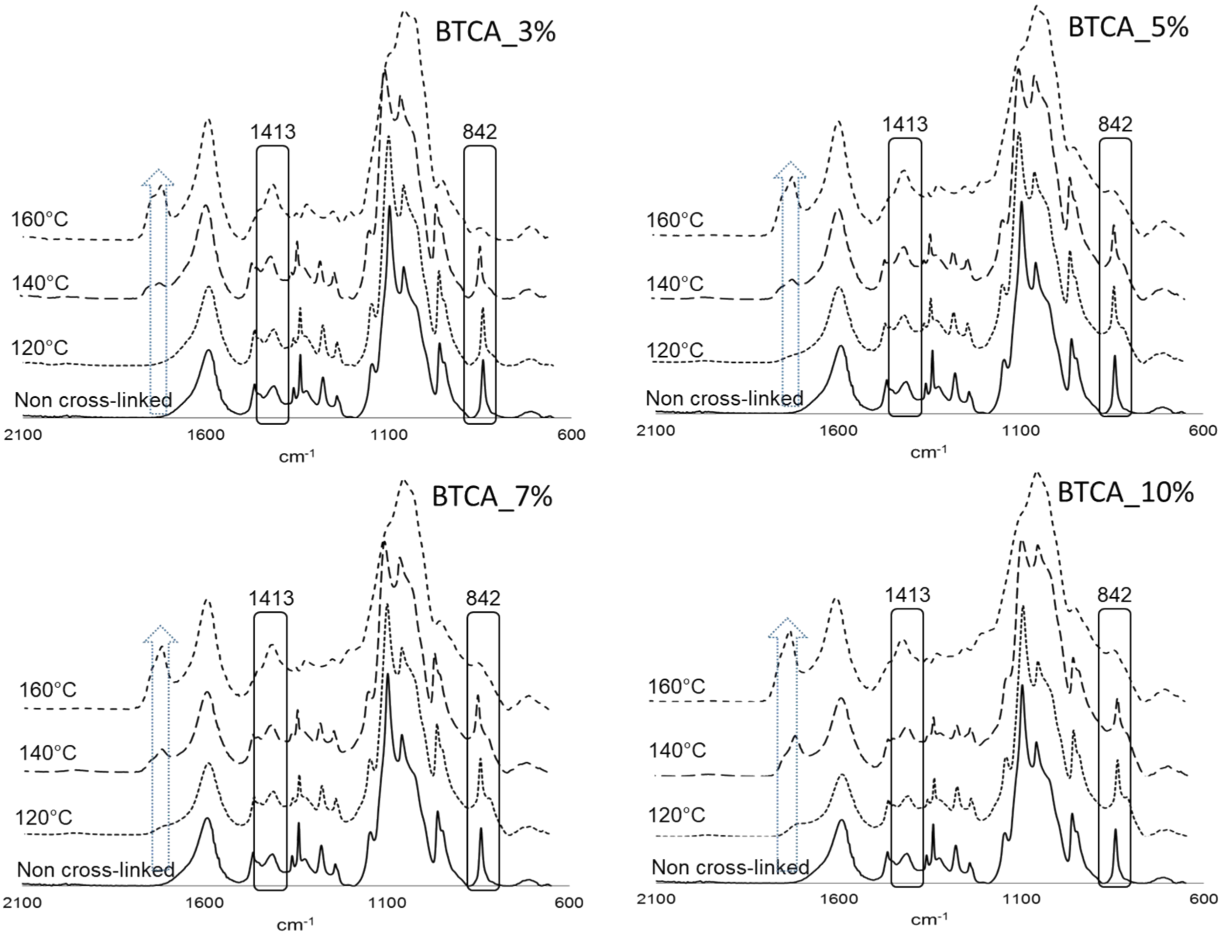

Fig. S3 FTIR spectra of absorption bands in 600 to 2100 cm^{-1} region (changes in the intensity of absorption bands 1420 cm^{-1} and 840 cm^{-1}).

Contact angle measurements

The wettability of electrospun samples was determined using a OCA15+ goniometer system (Dataphysics, Germany) with the sessile drop method. Static contact angle (SCA) measurements were carried out using ultra-pure water at ambient temperature. All measurements were carried out on at least two independent surfaces with a drop volume of 2 μL. Each SCA value was the average of at least five drops of liquid per surface.

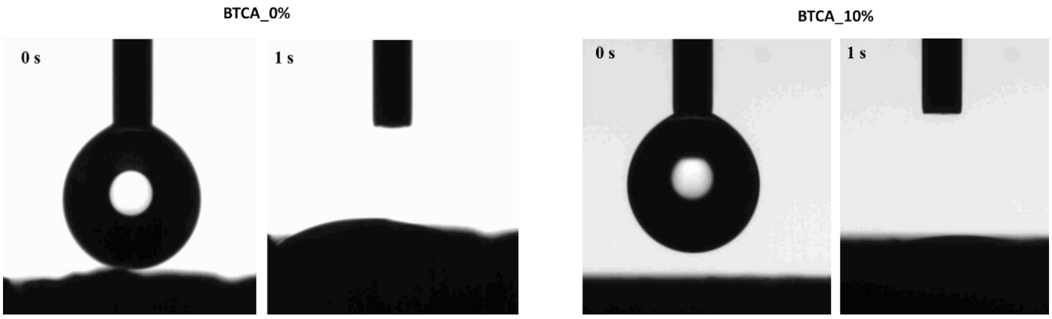

Fig. S4 Static water contact angel images of CMC/PEO electrospun nanofiber samples cross-linked with 0 and 10 wt.% BTCA at 160 ° C

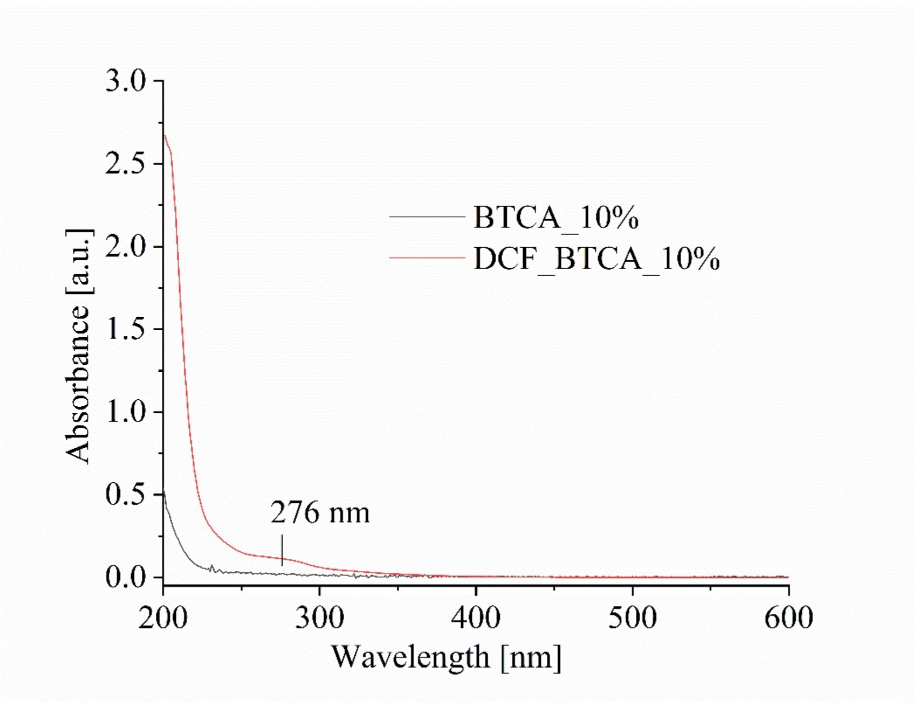

Fig. S5 UV-Vis spectra of DCF incorporated electrospun samples cross-linked with 10 wt.% BTCA at 160 °C.

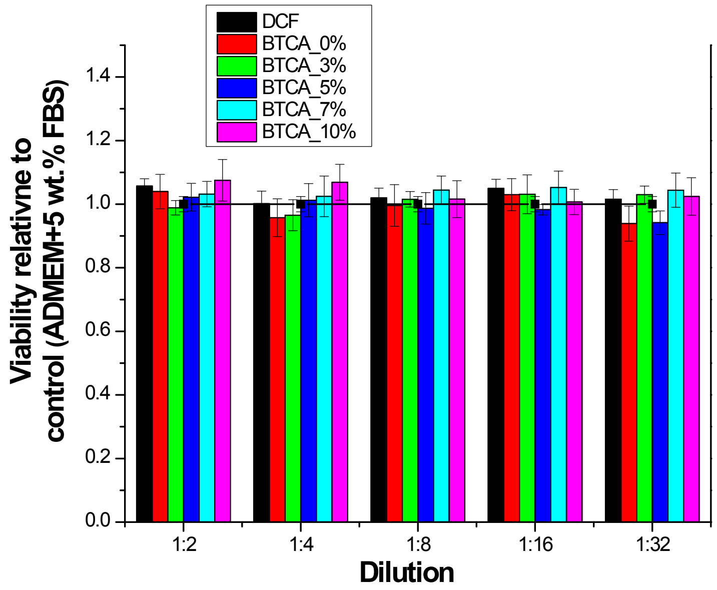

Figure S6. Viability of human skin derived fibroblasts at different dilusions after exposure to the DCF incorporated electrospun nanofibrous mats cross-linked with different concentrations of BTCA at 160 °C. The shown results were calculated relative to control (pure cell growth media). The black line correspond to the calculated confidence intervals for the control sample.
